# Reversible data hiding for 3D mesh models based on spatial polygon prediction and dual sorting

**DOI:** 10.1038/s41598-026-47050-3

**Published:** 2026-04-09

**Authors:** Qingjun Wang, Qilong Zhang, Xiaoying Song, Yiwen Liu

**Affiliations:** 1 School of Artificial Intelligence, Dalian Neusoft University of Information, Dalian, 116023 China; 2 Liaoning Provincial Key Laboratory of Network Security and Computing Technology, Dalian Neusoft University of Information, Dalian, 116023 China; 3 School of Information Science and Engineering, Northeastern University, Shenyang, 110819 China

**Keywords:** Reversible data hiding, 3D mesh models, Prediction, Dual sorting, Engineering, Mathematics and computing

## Abstract

Reversible data hiding (RDH) is a crucial information hiding technique for copyright protection and integrity verification of digital media, as it ensures the original content can be perfectly restored. However, current research on RDH remains predominantly focused on images, and relatively little attention has been paid to three-dimensional (3D) models. To address this gap, this paper proposes a RDH method for 3D mesh models based on spatial polygon prediction and dual sorting. First, a three-layer vertex division mechanism is introduced to enable a two-round embedding process, thereby expanding the number of embeddable vertices and increasing embedding capacity. Then, a spatial polygon prediction scheme is designed to balance the dual objectives of enhancing prediction accuracy and preserving embedding capacity. Furthermore, a dual sorting strategy that jointly considers angular smoothness and edge-length regularity is proposed to prioritize the smoother embedding units for data embedding, leveraging their smaller prediction errors to reduce geometric distortion. Finally, secret data is reversibly embedded into 3D mesh models using a prediction error expansion (PEE) technique guided by the dual sorting. The experimental results demonstrate that the proposed method achieves a better balance between high embedding capacity and low geometric distortion compared with other state-of-the-art spatial-domain RDH methods.

## Introduction

Reversible data hiding (RDH) has garnered substantial research interest for its ability to embed secret data (e.g., digital signatures, copyright marks, or metadata) into a cover medium while ensuring the original content can be perfectly restored after extraction^1^. This reversibility is particularly critical in sensitive domains—such as medical imaging, military communications, digital archiving, and forensic analysis—where any permanent alteration is impermissible, and RDH thus enables reliable copyright protection, integrity verification, and content annotation^2–4^. Meanwhile, with the rapid advancement of multimedia technology and computer graphics, 3D models in the form of meshes or point clouds are being widely adopted across various fields, including industrial production, medical applications, film production, computer-aided design, and the video game industry^5–7^. Consequently, RDH for 3D models has attracted growing attention within the data hiding community, highlighting a pressing need for effective and reversible protection mechanisms tailored to these complex digital assets.

While RDH for digital images has been extensively studied for decades, with performance approaching theoretical limits, extending RDH to other digital media like audio, video, and especially 3D models remains at a preliminary stage. Research specifically addressing 3D models is particularly limited, owing to the challenges posed by their complex topological structures and irregular geometry. To address these challenges, existing RDH methods for 3D models have been explored primarily along four technical directions: spatial domain^8–15^, transform domain^16^, compressed domain^17–19^, and encrypted domain^20–27^. Spatial domain methods directly modify the vertex coordinates of 3D models to embed secret data with minimal geometric distortion. Transform domain methods operate by converting the original model into a representation within a selected transform domain and then embedding the secret data into transform coefficients. Compressed domain methods use compression algorithms such as vector quantization and progressive compression to compress models for data embedding. Encrypted domain methods first encrypt the model and then embed secret data into the encrypted domain to fulfill the privacy protection requirements in cloud environments.

In recent years, significant progress has been made in spatial-domain 3D RDH, and several typical embedding techniques such as difference expansion (DE), histogram shifting (HS), and prediction error expansion (PEE) have been proposed. Wu and Wang^8^ proposed a RDH method for 3D mesh models based on the difference expansion and difference shifting, which embeds secret data into the vertex coordinates by modifying the differences between the adjacent vertex coordinates. Although this method achieves high embedding capacity, it introduces obvious geometric distortion. Huang et al.^9^ proposed a RDH method for 3D mesh models based on HS, which constructs histogram using the normalized distance differences between the neighboring vertices. Gridhar and Kumar^10^ embedded secret data by shifting differences between vertices and employed a chaotic logistic map to decide the coordinate for embedding. Wu and Dugelay^11^ first extended the classical image PEE technique to 3D meshes. In their approach, the position of a target vertex is predicted by calculating the centroid of its traversed neighbors, and then the prediction error is expanded to embed data. By leveraging the context of local neighboring vertices to generate prediction errors (PEs), this PEE framework typically achieves superior embedding performance, and has thus served as a foundation for many subsequent advancements.

Building upon the PEE framework, recent research has focused on optimizing its core components—the vertex prediction scheme and the embedding order selection. For instance, Jiang et al.^12^ proposed a RDH algorithm for 3D mesh models based on the optimal three-dimensional prediction-error histogram (PEH) modification with the recursive construction coding. However, their odd–even prediction scheme utilizes only half of the neighboring vertices (selected by odd or even indices) as references to predict central vertex, which limits prediction accuracy. Zhang et al.^13^ first proposed an improved RDH method for 3D mesh models based on PEE and sorting. Their approach uses a ring prediction scheme to generate PEs and minimizes geometric distortion by optimizing the prediction error sequence. Zhang et al.^14^ introduced a hybrid prediction scheme and a multilayer strategy with overlapping partition to improve performance. However, more geometric distortion is introduced due to repeated data embedding at the same vertices. Shah et al.^15^ first proposed a novel multilayer perceptron based predictor for more accurate vertex predictions, but at the cost of high computational complexity and low embedding capacity.

As outlined above, the core challenge in RDH lies in balancing two competing objectives: high embedding capacity versus low distortion. To achieve a superior capacity-distortion trade-off, this paper proposes a spatial-domain RDH method for 3D mesh models based on spatial polygon prediction and dual sorting. First, the vertices of a 3D mesh model are divided into a candidate set and a reference set through a three-layer vertex division mechanism. Then, a spatial polygon prediction scheme is designed to generate the PEs by leveraging the high geometric similarity between neighboring vertices. Furthermore, a dual sorting strategy based on both angular smoothness and edge-length regularity is proposed. This strategy evaluates these two metrics to prioritize the smoother embedding units for data hiding. Finally, the integration of dual sorting with PEE technique is employed to maximize embedding capacity and minimize geometric distortion of marked mesh models. Experimental results demonstrate that the proposed method outperforms the existing conventional PEE method^11^, the PEH modification method^12^, and the PEE based on sorting method^13^. The main contributions of this paper are summarized as follows.

1. A three-layer vertex division mechanism is proposed to enable a two-round embedding process. By alternately partitioning vertices into candidate and reference sets, this mechanism effectively increases the number of embeddable vertices, overcoming the embedding capacity limitations of conventional single-round or fixed-partition strategies and thereby achieving higher embedding capacity.
2. A spatial polygon prediction scheme is designed to enhance the balance between prediction accuracy and embedding capacity. This scheme constructs spatial polygons to more fully exploit the geometric correlations among neighboring vertices, achieving more accurate prediction while maintaining reversibility.
3. A dual sorting strategy is introduced to effectively reduce geometric distortion. This strategy simultaneously considers two complementary metrics—angular smoothness and edge-length regularity—to prioritize geometrically smoother embedding units for data hiding. Compared with existing methods that rely on a single sorting metric, this strategy more precisely targets regions with smaller prediction errors, thereby significantly reducing the geometric distortion of marked mesh models.

The remainder of this paper is organized as follows. Section “Proposed method” introduces the key components of the proposed method, including mesh preprocessing, three-layer vertex division, spatial polygon prediction, dual sorting, data embedding and extraction. Section “Implementation of the proposed method” elaborates on the implementation of the proposed method. Section “Experimental results and analysis” analyzes the experimental results. Section “Discussion and potential extensions” presents a discussion of the proposed method and point out its potential extensions, and Section “Conclusions” concludes the paper.

## Proposed method

### Preprocessing

A 3D mesh model comprises vertex data and face data. The mesh geometry is represented by a vertex set, denoted by *V* = {**v**_1_, **v**_2_, …**v**_N_}, where a vertex position **v**_*i*_ specifies the coordinates $({v}_{i,x}, {v}_{i,y}{, v}_{i,z})$ in ${R}^{3}$. Typically, the vertex coordinates are stored as decimal format, with each coordinate ${v}_{i,k}$ (where $k\in \{x, y, z\})$ satisfying ${v}_{i,k}$<1. Many common 3D mesh formats like PLY, OBJ, and OFF employ an indexed data structure, where each vertex is assigned a unique index and connected to others according to the mesh topology. The mesh model connectivity is described by a face set, denoted as $F=\{{f}_{1}{,f}_{2},...{f}_{M}\}$ and each face is consisting of three vertices. Two vertices are defined as neighbors if they are connected by an edge. Thus, the vertices belonging to the same face are co-neighbors. The reversible data hiding method proposed in this paper is not only designed for manifold triangular meshes but can also be naturally extended to polygonal manifold meshes.

Vertex coordinates are typically represented by a 32-bit decimal number with 6-digit precision. However, such high precision is often unnecessary for most applications, allowing these coordinates to be lossily quantized into *p* levels^28^. Following the approaches in^12,13^, we employ a truncation function, denoted as $\left\lfloor \cdot \right\rfloor$ to convert decimal coordinates into integers, with the parameter *p* set to 4. Specifically, prior to prediction, all vertex coordinates are scaled by 10^*p*^, and the fractional parts are truncated. The integer coordinate $v_{i,k}^{{\mathrm{int}}}$ of vertex *i* is given by

1 $$v_{i,k}^{{\mathrm{int}}} = \left\lfloor {v_{i,k} \times 10^{p} } \right\rfloor , \, k \in \{ x,y,z\}$$

where $v_{i,k}^{{}}$ is the decimal coordinate of vertex *i*, and $k \in \{ x,y,z\}$ denotes the specific coordinate axis being processed. In the proposed method, both vertex prediction and data embedding rely on integer arithmetic. After data embedding, the modified integer coordinate $\tilde{v}_{i,k}^{{\mathrm{int}}}$ of the marked 3D mesh model is obtained. Then, the decimal coordinate $g_{i,k}$ transformed back from $\tilde{v}_{i,k}^{{\mathrm{int}}}$ is computed by

2 $$g_{i,k} = \tilde{v}_{i,k}^{{\mathrm{int}}} /10^{p} , \, k \in \{ x,y,z\}$$

where $\tilde{v}_{i,k}^{{\mathrm{int}}}$ is the modified integer coordinate of vertex *i*, and $k \in \{ x,y,z\}$ denotes the specific coordinate axis being processed.

To guarantee reversibility, the vertices of the 3D mesh model *M* are partitioned into two independent sets: the candidate set *C* and the reference set *R*. Set *C* comprises the vertices selected as candidates for data embedding, while set *R* provides reference vertices for prediction. Crucially, the two sets are mutually independent, modification to candidate vertices in *C* does not affect those reference vertices in *R*. This independence property is essential for reversibility, as it guarantees that the prediction values remain unchanged during both data embedding and extraction.

### Three-layer vertex division

For the mesh model *M*, let *D* be its complete vertex set. The division of *D* into candidate set *C* and reference set *R* is crucial for reversibility and embedding capacity. Since the prediction scheme necessitates a substantial reference set *R*, the candidate set *C* should be correspondingly limited, which constrains the embedding capacity. Therefore, expanding the number of candidate vertices is key to enhancing the embedding capacity. Based on this consideration, we proposed a three-layer vertex division method which can enables a two-round embedding strategy to increase the embedding capacity. The two-round embedding strategy involves an alternative usage of vertices in set *C* and set *R*, which can nearly double the embedding capacity. The three-layer vertex division method and two-round embedding strategy is detailed below.

First, we divide all the vertices in set *D* into three disjoint sets: *C*_1_, *C*_2_, and *D*_r_. This is achieved by performing a modulo-3 operation on the index value of each vertex in set *D*. Vertices with a remainder of 0 are assigned to set *C*_1_, those with a remainder of 1 to *C*_2_, and all remaining vertices are placed into set *D*_r_.

**Fig. 1:**
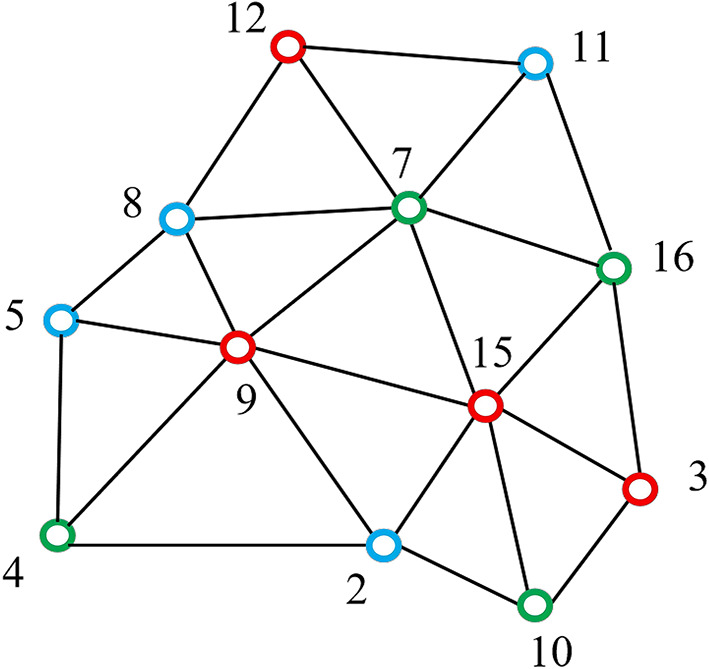
An example of three-layer vertex division.

Then, the payload *P* is partitioned into two sub-payloads with similar sizes *P*_1_ and *P*_2_ for the two embedding rounds. In the first round, the candidate and reference sets are assigned as *C* = *C*_1_ and *R* = *D*_r_ ∪ *C*_2_, respectively. *P*_1_ is embedded by modifying the coordinates of candidate vertices in *C*_1_, while the reference vertices in *R* remain unchanged to support prediction. In the second round, we set *C* = *C*_2_ and *R* = *D*_r_ ∪ *C*_1_, and *P*_2_ is then embedded into set *C*_2_. Note that the reference set *R* in this round includes the modified vertices of *C*_1_ during the first embedding round. As illustrated in Fig. 1, the red dots {3, 9, 12, 15} represent vertices in *C*_1_, the green dots {4, 7, 10, 16} represent vertices in *C*_2_, and the blue dots {2, 5, 8, 11} represent vertices in *D*_r_. For example, in the first embedding round, the position of vertex 9 (a red dot) is predicted using the reference vertices belonging to set *R* within its 1-ring neighbourhood, including {2, 5, 8} (blue dots) and {4, 7} (green dots). Similarly, in the second round, position of vertex 7 is predicted by its adjacent reference vertices, which include {8, 11} and {9, 15, 12}.

In summary, the proposed three-layer vertex division enables the alternative use of vertices between candidate set *C* and reference set *R*. This strategy increases the number of available vertices for embedding data, which significantly increases the embedding capacity.

### Spatial polygon prediction

The strong geometric correlation between local neighboring vertices enables high predictability of vertex positions within the 1-ring neighborhood. Currently, there are two widely adopted prediction schemes to generate a sharp prediction error histogram: odd–even prediction scheme^12,23^ and ring prediction scheme^13,22^. The old-even prediction scheme uses only half of the 1-ring neighbors for central vertex prediction based on index parity, which constrains the prediction accuracy. Conversely, the ring prediction scheme employs all 1-ring neighbors as reference vertices for prediction to improve prediction accuracy. However, since all neighbors are reserved for prediction, fewer vertices remain available for embedding data, thereby limiting the embedding capacity. To better balance prediction accuracy and embedding capacity, this paper proposes a spatial polygon prediction scheme that uses approximately two-thirds of the 1-ring neighbors to predict the central vertex as a compromise. We first introduce two definitions for the spatial polygon as follows.

#### Definition 1.

 Spatial polygon. A spatial polygon is defined by a finite set of distinct points A_1_, A_2_, …, A_n_ (where n ≥ 3) in 3D space. These points are connected sequentially by straight-line segments to form a closed spatial polygonal chain.

#### Definition 2.

Interior angle of a spatial polygon. In plane geometry, the interior angle of a polygon is the angle formed between two adjacent sides. For a non-planar spatial polygon, the interior angle at a given vertex is the angle in [0, π) formed by its two incident edges within the plane which they uniquely define.

**Fig. 2:**
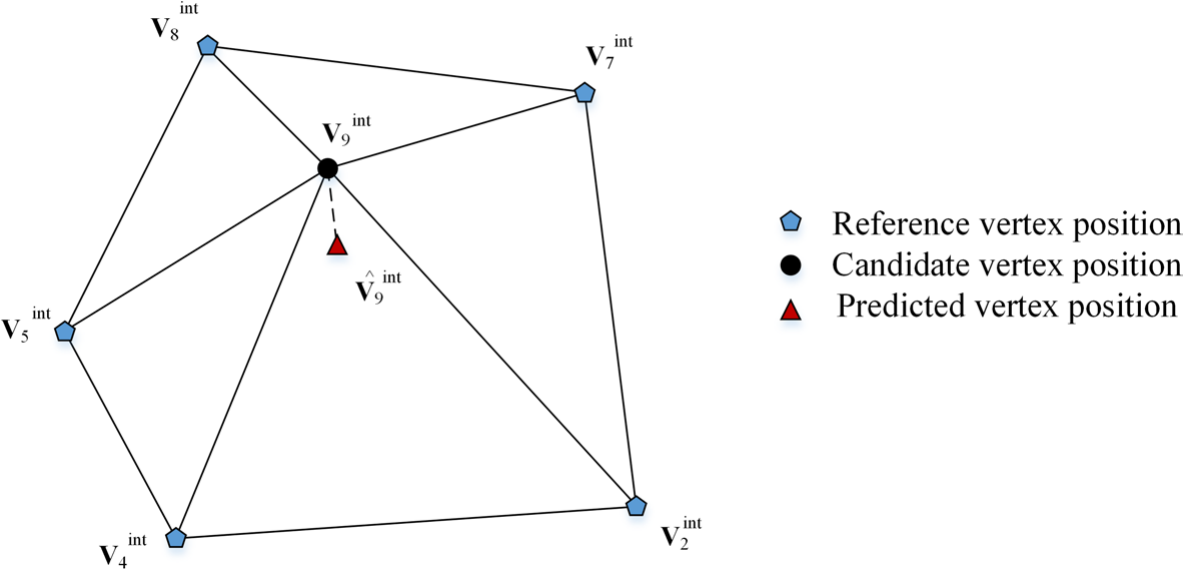
An example of vertex position prediction in an embedding unit.

Then, an embedding unit is defined as a central vertex from set *C* and its associated spatial polygon. The spatial polygon is formed by the *n* reference vertices belonging to *R* in the central vertex’s 1-ring neighborhood. In an embedding unit, the central vertex is modified to embed data, while the vertices of the spatial polygon serve as the prediction context and remain unchanged. Figure 2 shows an example of vertex position prediction using the spatial polygon prediction scheme. The embedding unit comprises central vertex 9 and its neighboring reference vertices {2, 4, 5, 7, 8}, the later set forming a spatial polygon. The position of central vertex 9 is derived from the vertices of the spatial polygon. Embedding units are constructed by traversing vertices in set* C* in ascending index order. For each vertex *i* ∈ *C*, its position ${\hat{\mathbf{v}}}_{i}^{{\mathrm{int}}}$ is predicted using the vertices of its associated spatial polygon as

3 $${\hat{\mathbf{v}}}_{i}^{{\mathrm{int}}} = \frac{1}{{N_{i} }}\sum\limits_{j = 1}^{{N_{i} }} {{\mathbf{v}}_{j}^{{\mathrm{int}}} }$$

where ${N}_{i}$ is the number of spatial polygon vertices for vertex *i*, and ${\hat{\mathbf{v}}}_{i}^{{\mathrm{int}}}$ is the predicted vertex position. During the data embedding, vertices in the reference set* R* are not modified, so the predicted vertex position ${\hat{\mathbf{v}}}_{i}^{{\mathrm{int}}}$ keeps unchanged. Based on the real vertex position ${\mathbf{v}}_{i}^{{\mathrm{int}}}$ and the predicted vertex position ${\hat{\mathbf{v}}}_{i}^{{\mathrm{int}}}$, the prediction-error vector **e**_*i*_ is computed as

4 $${\mathbf{e}}_{i}^{{}} = {\mathbf{v}}_{i}^{{\mathrm{int}}} - {\hat{\mathbf{v}}}_{i}^{{\mathrm{int}}} = (v_{i,x}^{{\mathrm{int}}} - \hat{v}_{i,x}^{{\mathrm{int}}} ,v_{i,y}^{{\mathrm{int}}} - \hat{v}_{i,y}^{{\mathrm{int}}} ,v_{i,z}^{{\mathrm{int}}} - \hat{v}_{i,z}^{{\mathrm{int}}} )$$

where $(v_{i,x}^{{\mathrm{int}}} ,v_{i,y}^{{\mathrm{int}}} ,v_{i,z}^{{\mathrm{int}}} )$ and $(\hat{v}_{i,x}^{{\mathrm{int}}} ,\hat{v}_{i,y}^{{\mathrm{int}}} ,\hat{v}_{i,z}^{{\mathrm{int}}} )$ are the integer coordinates of ${\mathbf{v}}_{i}^{{\mathrm{int}}}$ and ${\hat{\mathbf{v}}}_{i}^{{\mathrm{int}}}$ in ${R}^{3}$, respectively.

Then, we calculate the prediction-error vectors of all embedding units to generate the PEs. The geometric similarity among local neighboring vertices leads to small PEs, which are suitable for data embedding. The proposed spatial polygon prediction scheme achieves a better trade-off between prediction accuracy and capacity. It increases the number of neighboring reference vertices over the odd–even prediction scheme for better prediction accuracy, while also freeing up more vertices for data embedding compared to the ring prediction scheme for higher embedding capacity.

### Dual sorting

In image-based RDH, sorting serves as a pivotal strategy for enhancing embedding performance by optimizing the order of PEs. However, most existing RDH algorithms for 3D meshes neglect the critical impact of the embedding sequence on both embedding capacity and visual quality. Zhang et al.^13^ first proposed a sorting-based PEE method for 3D mesh models, achieving a significant performance improvement over previous methods. By utilizing mean curvature as a smoothness metric, their method prioritizes smooth regions to embed data, as vertex positions there can be accurately predicted, resulting in smaller prediction errors. Building on this insight, we propose a dual sorting strategy that evaluates the smoothness of an embedding unit from two complementary perspectives: angular smoothness and edge-length regularity of the spatial polygon. Smoother embedding units are assigned high priority for data embedding, thus reducing geometric distortion.

#### Primary sorting: angular smoothness

The first criterion is based on the geometric principle that a spatial polygon with *n* vertices becomes smoother as the sum of its internal angles approaches the planar sum of $(n-2)\times \pi$. Thus, the angular smoothness (*AS*) of the spatial polygon for the candidate vertex *i* is given by

5 $$AS(i) = |1 - \frac{{\sum\nolimits_{j = 1}^{{N_{i} }} {\theta_{j} } }}{(n - 2) \times \pi }|$$

where ${\theta }_{j}$ is the internal angle at the *j*-th vertex and *N*_*i*_ is the number of vertices in the spatial polygon for vertex *i*. The closer the *AS* value is to 0, the smoother the spatial polygon. We sort all embedding units according to their *AS* values in ascending order to form a primary sequence, which is divided into *L* contiguous subsequences.

#### Secondary sorting: edge-length regularity

The second criterion evaluates the spatial polygon’s geometric regularity based on edge-length variance. A small variance indicates a more regular geometric shape, which corresponds to less visual roughness. The edge-length regularity (*ELR*) of the spatial polygon for the candidate vertex *i* is calculated as

6 $$ELR(i) = \frac{1}{{N_{i} }}\sum\limits_{j = 1}^{{N_{i} }} {(||{\mathbf{v}}_{j} - {\mathbf{v}}_{(j + 1)\bmod n} || - \overline{m}_{i} )^{2} }$$

where $\Vert \cdot \Vert$ denotes the Euclidean distance between two vertices, which corresponds to the length of the edge connecting them, *N*_*i*_ is the number of vertices in the spatial polygon for vertex *i*, **v**_*j*_ is the position coordinate of the *j*-th vertex of spatial polygon, and the mean edge length $\overline{m}_{i}$ is given by

7 $$\overline{m}_{i} = \frac{1}{{N_{i} }}\sum\limits_{j = 1}^{{N_{i} }} {||{\mathbf{v}}_{j} - {\mathbf{v}}_{(j + 1)\bmod n} ||}$$

#### Dual sorting parameter

For the candidate vertex *i*, the dual sorting parameter (*DSP*), integrating the two aforementioned geometric criteria to determine the final embedding sequence, is defined as

8 $$DSP(i) = C_{l} + ELR(i) \times L$$

where *C*_*l*_ = *l* (with 1 ≤ *l* ≤ *L*) and vertex *i* belongs to the *l*-th subsequence from the primary sequence.

The parameter *L* plays a dual role in the sorting process. Based on experimental results, *L* is set to 4, which represents a robust empirical choice that balances the influence of both criteria. On one hand, it is used to partition the primary sequence into several subsequences for computation during dual sorting. On the other hand, the value of *L* determines the relative weight of angular smoothness (*AS*) and edge-length regularity (*ELR*) in the final sorting. If *L* is too small, the contribution of *AS* becomes excessively low, causing the dual sorting process to degenerate into an approximately single-criterion sorting. Conversely, if *L* is too large, *AS* dominates the ranking, diminishing the role of *ELR* information.

The *DSP* value has two key features. First, this value remains unchanged after data embedding. Second, a smaller *DSP* value indicates an embedding unit with superior local smoothness, and vice versa. Since vertex positions in smooth embedding units can be predicted more accurately, the prediction errors typically exhibit smaller magnitudes. All embedding units of the mesh model are sorted in ascending order of their *DSP* values, with units having smaller *DSP* values being more suitable for data hiding. Thus, the embedding process starts from the unit with the smallest *DSP* value in the sorted list and proceeds to the next embedding units until all data bits are embedded. As the *DSP* values are preserved throughout both data embedding and extraction, the receiver can reconstruct the same sorted order, which is essential for ensuring reversibility.

### Data embedding and extraction

After spatial polygon prediction and dual sorting, the sorted PE sequence ${E}_{sort}$ is obtained. Assume that ${E}_{sort}=\{{e}_{1,x},{e}_{1,y},{e}_{1,z},...{e}_{m,x},{e}_{m,y},{e}_{m,z}\}$, where *m* is the number of the embedding units. Data embedding and extraction are performed directly on ${E}_{sort}$ by modifying the prediction errors. For recovering data, the auxiliary information should be known first. The auxiliary information comprises four parameters: threshold values ${T}_{l}$ (18 bits) and *T*_*r*_ (18 bits), subsequences parameter* L* (4 bits), and payload size |*P*_1_| or |*P*_2_| (24 bits), totaling 64 bits. To ensure correct extraction, the auxiliary information must be embedded before the payload in each embedding round. Before data embedding, both the payload and auxiliary information are encoded as bitstreams. The data embedding and extraction processes are detailed below.

#### Auxiliary information embedding and extraction

Auxiliary information is embedded using LSB replacement method. The LSB values of the first 64 prediction errors in ${E}_{sort}$ are read to form a binary sequence S_*LSB*_, which is then prepended to the payload. The LSB value of the original prediction error ${e}_{i,k}$ is computed as

9 $$\begin{array}{*{20}c} {{\mathrm{LSB(}}e_{i,k}^{{}} {)} = e_{i,k}^{{}} - 2\left\lfloor {e_{i,k}^{{}} {/}2} \right\rfloor {,}} & {k \in \{ x,y,z\} } & {} \\ \end{array}$$

The LSB of ${e}_{i,k}$ is then replaced by auxiliary information bit $b \in \{ 0,1\}$_,_ and the modified prediction error $\tilde{e}_{i,k}^{{}}$ is calculated as

10 $$\begin{array}{*{20}c} {\tilde{e}_{i,k}^{{}} { = 2}\left\lfloor {e_{i,k}^{{}} /2} \right\rfloor + b{,}} & {k \in \{ x,y,z\} } \\ \end{array}$$

During extraction, the embedded bit *b* is extracted by

11 $$\begin{array}{*{20}c} {b = \tilde{e}_{i,k}^{{}} - 2\left\lfloor {\tilde{e}_{{i{,}k}}^{{}} {/}2} \right\rfloor ,} & {k \in \{ x,y,z\} } & {} \\ \end{array}$$

Using the corresponding bit from S_*LSB*_, the original prediction error *e*_*i,k*_ is recovered by

12 $$\begin{array}{*{20}c} {e_{i,k}^{{}} { = 2}\left\lfloor {\tilde{e}_{i,k}^{{}} /2} \right\rfloor + {\mathrm{LSB(}}e_{i,k}^{{}} {)},} & {k \in \{ x,y,z\} } & {} \\ \end{array}$$

#### Payload embedding and extraction

The payload is embedded into prediction errors using PEE method. The process of embedding one bit into a prediction error (e.g.,${e}_{i,x}$ for vertex *i*) is described below; the same procedure applies to ${e}_{i,y}$ and ${e}_{i,z}$. Starting from the 65-th prediction error in ${E}_{sort}$, each original prediction error can be expanded or shifted to embed data, and the modified prediction error $\tilde{e}_{{i{,}x}}^{{}}$ is is calculated as

13 $$\tilde{e}_{{i{,}x}}^{{}} = \left\{ {\begin{array}{*{20}l} {2e_{{i{,}x}} + b,} \hfill & {{\mathrm{if}}\,e_{{i{,}x}}^{{}} \in [T_{l} {,}T_{r} )} \hfill \\ {e_{{i{,}x}} + T_{r} ,} \hfill & {{\mathrm{if}}\,e_{{i{,}x}}^{{}} \in [T_{r} {,} + \infty )} \hfill \\ {e_{{i{,}x}} + T_{l} ,} \hfill & {{\mathrm{if}}\,e_{{i{,}x}}^{{}} \in ( - \infty {,}T_{l} )} \hfill \\ \end{array} } \right.$$

where $\tilde{e}_{{i{,}x}}^{{}}$ is the modified prediction error, ${T}_{l}$ and ${T}_{r}$ are the integer threshold values, and *b* is one bit of the payload. During extraction, *b* is extracted by

14 $$\begin{array}{*{20}c} {b = \tilde{e}_{{i{,}x}}^{{}} - 2\left\lfloor {\tilde{e}_{{i{,}x}}^{{}} {/}2} \right\rfloor ,} & {\begin{array}{*{20}c} {{\mathrm{if}}} & {\tilde{e}_{{i{,}x}}^{{}} \in [2T_{l} {,}\;2T_{r} )} \\ \end{array} } \\ \end{array}$$

The original prediction error ${e}_{i,x}$ is computed as

15 $$e_{{i{,}x}} = \left\{ {\begin{array}{*{20}l} {\left\lfloor {\tilde{e}_{{i{,}x}}^{{}} /2} \right\rfloor ,} \hfill & {{\mathrm{if}}\,\tilde{e}_{{i{,}x}}^{{}} \in [2T_{l} {,}2T_{r} )} \hfill \\ {\tilde{e}_{{i{,}x}} - T_{r} ,} \hfill & {{\mathrm{if}}\,\tilde{e}_{{i{,}x}}^{{}} \in [2T_{r} {, + }\infty )} \hfill \\ {\tilde{e}_{{i{,}x}} - T_{l} ,} \hfill & {{\mathrm{if}}\,\tilde{e}_{{i{,}x}}^{{}} \in {(} - \infty {,}2T_{l} {)}} \hfill \\ \end{array} } \right.$$

#### Appropriate threshold values

The embedding capacity is determined by the number of prediction errors within the interval [${T}_{l}$, ${T}_{r}$). An excessively large interval increases distortion, while a small one limits the embedding capacity. To balance capacity and distortion, an iterative search is performed to determine the suitable threshold values. The search starts with initial values ${T}_{l}$= − 1 and ${T}_{r}$= 0. The search terminates once the number of prediction errors within the interval [${T}_{l}$*,* ${T}_{r}$) is sufficient to accommodate the payload.

The search starts with initial values ${T}_{l}$= − 1 and ${T}_{r}$=0. This initialization is chosen because most prediction errors are concentrated near zero, allowing the smallest errors to be prioritized for embedding to minimize distortion. In each iteration, if the number of prediction errors within the current interval [${T}_{l}$, ${T}_{r}$) is insufficient to accommodate the required payload, the interval is expanded using a symmetric stepwise expansion strategy, preventing uneven distortion caused by unilateral expansion.

The search terminates when the number of prediction errors within the interval reaches or exceeds the required embedding capacity. The resulting ${T}_{l}$ and ${T}_{r}$ are then used as the threshold parameters for the current embedding round. Since the total number of prediction errors is finite and the count of embeddable errors within the interval is non-decreasing as the interval expands, the greedy search is guaranteed to terminate within a finite number of steps and find a feasible solution. If the required capacity exceeds the total number of prediction errors, the method cannot satisfy the payload demand; however, in the experiments conducted in this paper, all embedding capacities remain within the upper limit.

## Implementation of the proposed method

**Fig. 3:**
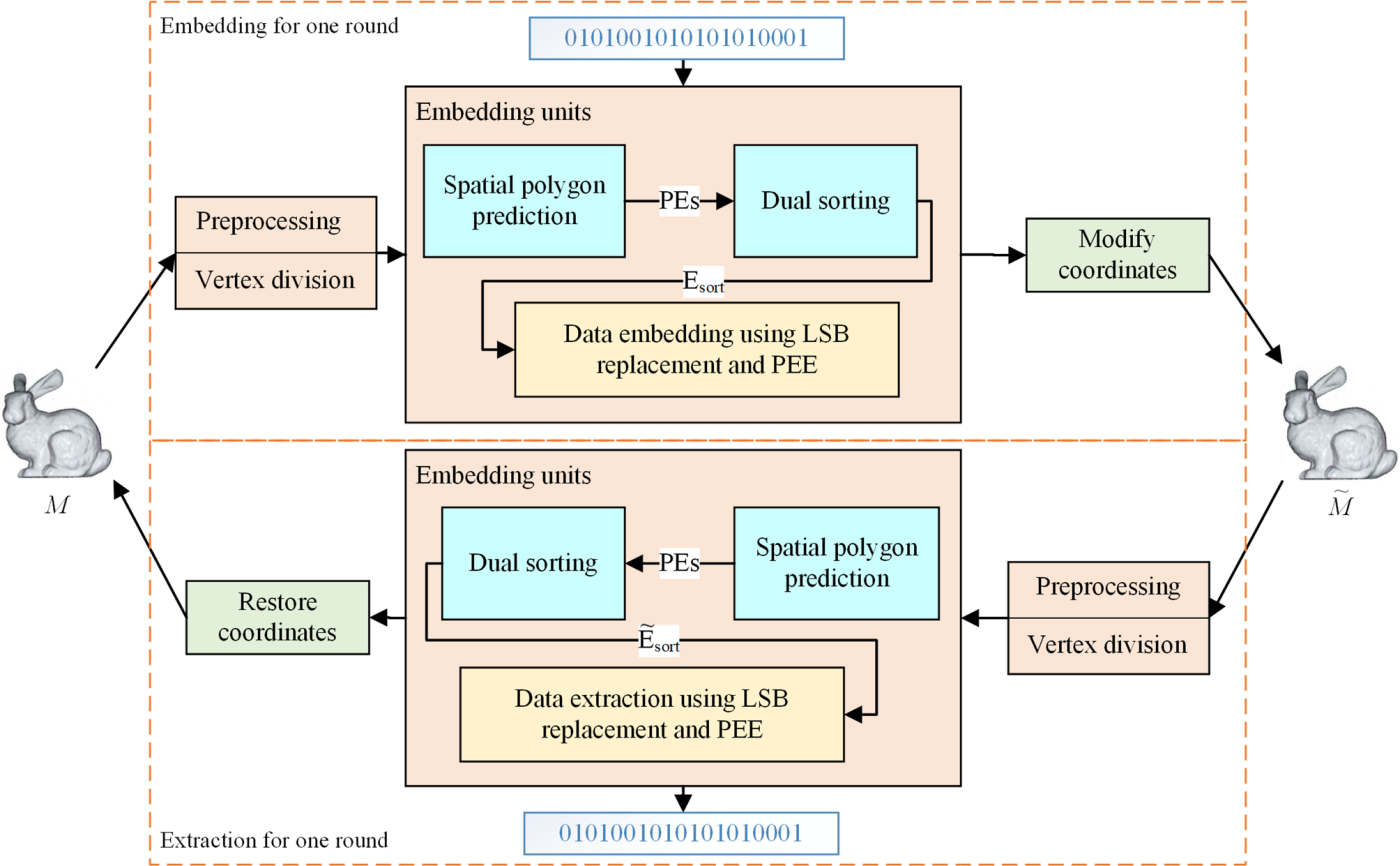
Framework of data embedding and extraction for one round.

This section details the implementation of the proposed RDH method for 3D mesh models. As previously outlined, the method embeds the payload *P* over two rounds, each handling approximately half of the payload (denoted as *P*_1_ or *P*_2_). The extraction procedure mirrors the embedding process in reverse: after both rounds, the complete payload *P* is retrieved and the original model *M* is perfectly recovered. Given the analogous nature of each round, we focus on illustrating a single round for clarity. The overall framework of the embedding and extraction procedures for one round is depicted in Fig. 3.

### Data embedding procedure

The model owner generates a marked model by embedding secret data into the original 3D mesh model through coordinate modification of the candidate vertices. The data embedding procedure for one round is designed as follows.

Step 1. Mesh preprocessing. Convert all vertex coordinates of the original mesh model from decimal into integer format.

Step 2. Three-layer Vertex division. Divide mesh vertices into three disjoint sets *C*_1_, *C*_2_ and *D*_r_. Set *C*_1_ or *C*_2_ is selected as the candidate set *C* for embedding data, while the other two sets constitute the reference set *R* used for prediction.

Step 3. Spatial polygon prediction. Construct all embedding units, each consisting of a central vertex from set *C* and its adjacent reference vertices from set *R*. For each embedding unit, calculate the predicted vertex position based on spatial polygon prediction scheme. Then, calculate the prediction-error vectors of all the embedding units to obtain the PEs.

Step 4. Dual sorting. Calculate the dual sorting parameter *DSP* for each embedding unit. Then, sort all embedding units in ascending order of *DSP* values to obtain the sorted PE sequence *E*_sort_.

Step 5. Data embedding. Find appropriate threshold values *T*_*l*_ and *T*_*r*_ through an iterative process to satisfy the target payload size |*P*_*1*_| or |*P*_*2*_|. Skip the first 64 elements in ${E}_{sort}$. Record the original 64 LSB values of prediction errors to obtain sequence *S*_*LSB*_ and include *S*_*LSB*_ to the payload *P*_1_ or *P*_2_. Replace the first 64 LSB values of the prediction errors by the auxiliary information. Then, embed the payload* P*_1_ or *P*_2_ into the rest prediction errors in ${E}_{sort}$ using the PEE method. Thus, obtain the modified PE sequence $\tilde{E}_{{{\mathrm{sort}}}}$.

Step 6. Marked mesh generation. For each vertex in set *C*, modify the coordinates by adding the prediction errors of $\tilde{E}_{{{\mathrm{sort}}}}$ to the corresponding predicted coordinates. Convert all the integer vertex coordinates back to decimal coordinates and then generate the marked mesh model $\tilde{M}$.

### Data extraction and model recovery procedure

The secret data can be extracted and the original mesh model *M* can be fully recovered by the receiver. Data extraction and model recovery procedure for one round is detailed as follows.

Step 1. Mesh preprocessing. Convert all vertex coordinates of the marked mesh from decimal into integer format.

Step 2. Three-layer vertex division. Divide the marked mesh vertices into three disjoint sets *C*_*1*_, *C*_*2*_ and *D*_*r*_. Set *C*_*1*_ or *C*_*2*_ is selected as the candidate set *C*, while the other two sets are selected as the reference set *R*. Note that if set *C*_*1*_ is first embedded, then set *C*_*2*_ is first extracted.

Step 3. Spatial polygon prediction. See Step 3 of the data embedding procedure. The same predicted vertex positions can be obtained on the receiver side. Compute the prediction-error vectors of all embedding units, then obtain the PEs.

Step 4. Dual sorting. Repeat the same Step 4 of embedding procedure to obtain the sorted PE sequence $\tilde{E}_{{{\mathrm{sort}}}}$.

Step 5. Data extraction. Read the first 64 LSB values from $\tilde{E}_{{{\mathrm{sort}}}}$ to recover the thresholds *T*_*l*_ and *T*_*r*_, payload size |*P*_*1*_| or |*P*_*2*_|, and subsequence parameter *L.* Skip the first 64 elements. Under the guidance of the extracted auxiliary information, inverse PEE operations are applied to the prediction errors in $\tilde{E}_{{{\mathrm{sort}}}}$. This process extracts the payload *P*_1_ or *P*_2_ and recover the original prediction errors. Then remove the sequence *S*_*LSB*_ from the payload *P*_1_ or *P*_2_. Replace the first 64 LSBs of the sorted prediction errors in $\tilde{E}_{{{\mathrm{sort}}}}$ with the original LSBs from *S*_*LSB*_. Thus, restore the whole original PE sequence ${E}_{sort}$.

Step 6. Recovery of the original mesh model *M*. For each vertex in candidate set *C*, restore the vertex coordinates by adding prediction errors in ${E}_{sort}$ to their predicted vertex positions. Then, convert all the integer vertex coordinates to decimal coordinates to recover the original mesh model *M*.

## Experimental results and analysis

The proposed method is implemented and tested in C +  + programming language under Windows 10 operating system. We use eight standard 3D mesh models with OBJ format in the experiment: Bunny, Horse, Casting, Crank, Venus, Dragon, Hand, and Dinosaur. These test models are selected from the datasets of Stanford 3D Scanning Repository and the 3D mesh watermarking benchmark provided by LIRIS. Table 1 shows the information of the eight experimental mesh models, which includes their numbers of vertices and faces. In the experiments, a randomly generated bitstream as the secret data is used, i.e., a 0/1 sequence generated by the C++ standard library function rand( ). The specific implementation uses a fixed seed to initialize the random number generator, ensuring the reproducibility of the experiments.

**Table 1:** Model information of the experiment.

Model	# vertices	# faces
Bunny	35,947	69,451
Horse	48,485	96,966
Casting	5,096	10,224
Crank	50,012	100,056
Venus	134,345	268,686
Dragon	437,645	871,414
Hand	36,619	72,958
Dinosaur	56,194	112,384

The embedding performance of the proposed method is compared with Wu et al.’s conventional PEE method^11^, Jiang et al.’s PEH modification method^12^ and Zhang et al.’s PEE with sorting method^13^. We mainly evaluate the performance of the proposed method with two metrics: the embedding rate (ER) and the signal-to-noise ratio (SNR). The key embedding capacity is measured by ER in bits per vertex (bpv), which represents the ratio of embedded bits to the total number of vertices. SNR quantifies the geometric distortion of the 3D mesh model, where a higher value corresponds to less distortion. SNR is defined as

16 $${\mathrm{SNR}} = 10\log_{10} \frac{{\sum\nolimits_{i = 1}^{N} {\left[ {(v_{i,x} - \overline{v}_{x} )^{2} + (v_{i,y} - \overline{v}_{y} )^{2} + (v_{i,z} - \overline{v}_{z} )^{2} } \right]} }}{{\sum\nolimits_{i = 1}^{N} {\left[ {(g_{i,x} - v_{i,x} )^{2} + (g_{i,y} - v_{i,y} )^{2} + (g_{i,z} - v_{i,z} )^{2} } \right]} }}$$

where $(v_{i,x} ,v_{i,y} ,v_{i,z} )$ are the original position coordinates of vertex *i*, $(\overline{v}_{x} ,\overline{v}_{y} ,\overline{v}_{z} )$ are the average position coordinates of the 3D mesh model, $(g_{i,x} ,g_{i,y} ,g_{i,z} )$ are the modified position coordinates of vertex *i*, and *N* is the number of vertices in the mesh model.

### Performance evaluation on experimental mesh models

**Fig. 4:**
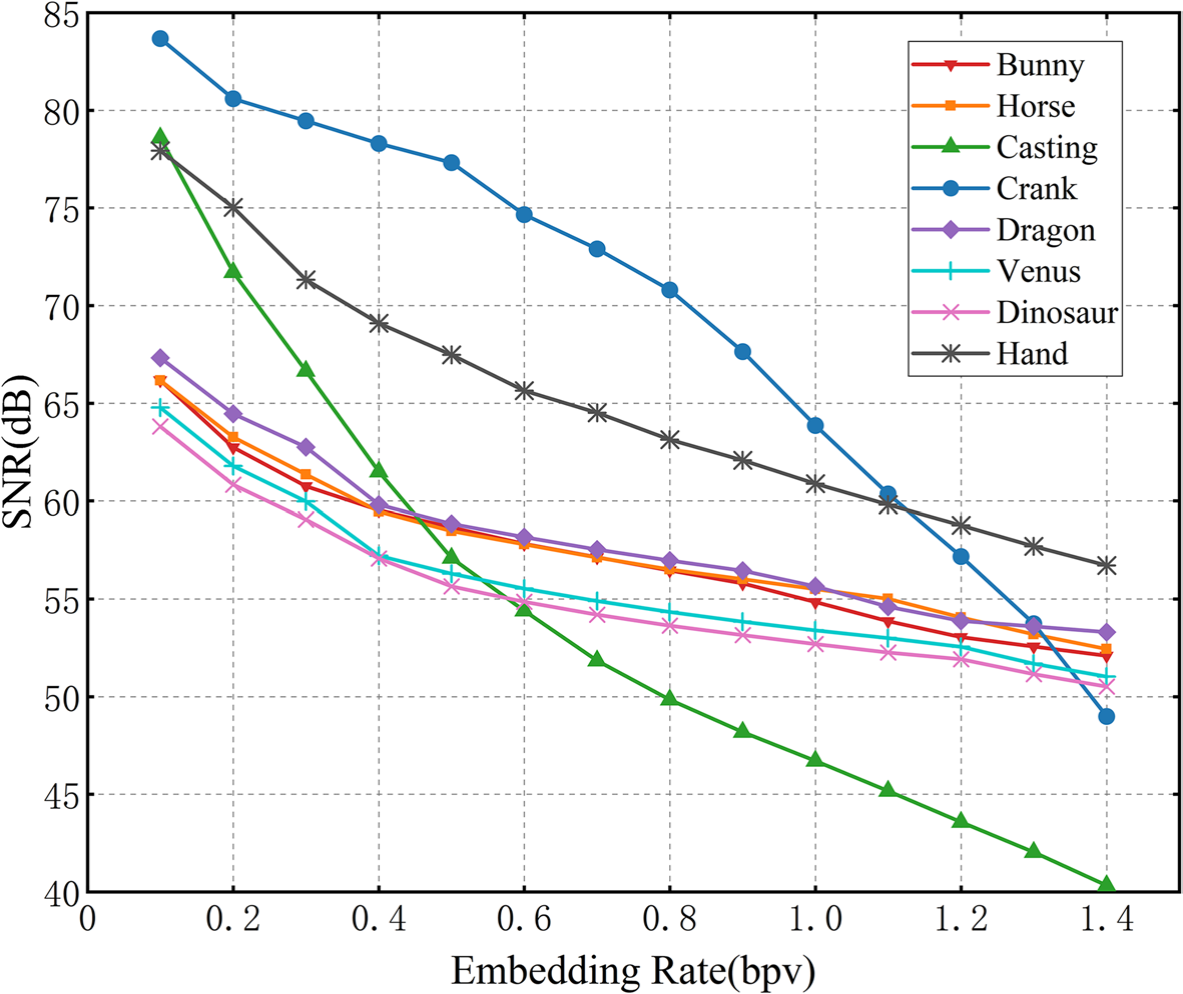
Performance curves of marked mesh models.

The SNR at different embedding rates on the experimental mesh models are shown in Fig. 4. As expected, SNR decreases with increasing embedding rate across all mesh models, reflecting the geometric distortion introduced during data embedding. Notably, all mesh models maintain an SNR above 50 dB at an embedding rate of 0.8 bpv, and even at 1.4 bpv, the SNR remains above 40 dB. The SNR decays more steeply in finer mesh models such as Casting, owing to their sparser distribution of vertices. Overall, an average SNR of 59.02 dB is achieved across all models. These results verify the effectiveness of the proposed method in achieving a balance between embedding capacity and visual quality.

The embedding capacity, embedding rate and SNR values of all experimental mesh models are summarized in Table 2. As noted, models with more vertices, such as the Dragon model, achieve a higher embedding capacity. Conversely, models such as the Horse attain a higher embedding rate because they require fewer reference vertices for candidate vertices prediction. Meanwhile, models with predominance of smooth regions (e.g., the Hand model) usually generate smaller prediction errors, resulting in a higher SNR. With an average embedding rate of 1.68 bpv across all mesh models, the results confirm the high embedding capacity of the proposed method.

**Table 2:** Performance evaluation of the proposed method on experimental mesh models.

Model	Capacity (bits)	ER (bpv)	SNR (dB)
Bunny	61,110	1.70	49.44
Horse	91,637	1.89	49.94
Casting	9020	1.77	37.43
Crank	81,019	1.62	30.06
Venus	213,609	1.59	50.38
Dragon	704,608	1.61	52.18
Hand	61,154	1.67	54.68
Dinosaur	88,225	1.57	49.97

### Visual effects of marked mesh models

**Fig. 5:**
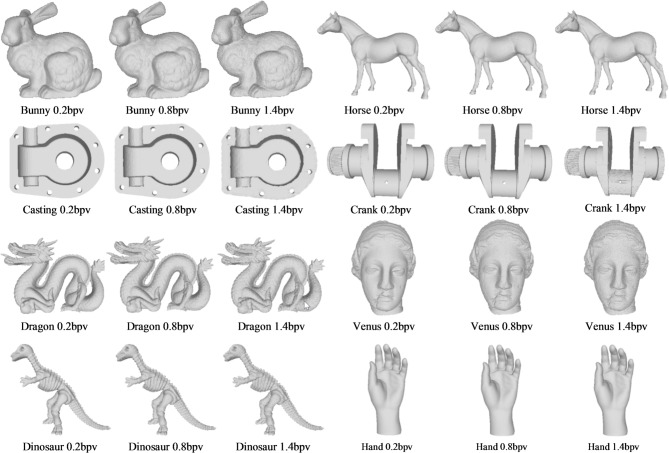
Visual effects of marked mesh models at different embedding rates.

Figure 5 illustrates the visual effects of the marked mesh models under different embedding rates. At low embedding rates, distortion in the marked mesh models is negligible. However, as the embedding rate increases, visible distortions become more apparent. Nevertheless, the proposed method limits visual distortion to an acceptable level even at the embedding rate of 1.40 bpv. Experimental results demonstrate that marked mesh models maintains high visual quality.

### Performance comparison with existing methods

The performance of the proposed method is compared with three state-of-the-art spatial-domain RDH methods: Wu et al.’s conventional PEE method^11^, Jiang et al.’s PEH modification method^12^ and Zhang et al.’s PEE with sorting method^13^. In^11^, data embedding is achieved by first predicting a vertex from its traversed neighbors and then expanding the prediction error for embedding. In^12^, the odd–even prediction scheme is employed to generate prediction errors. Meanwhile,^13^ utilizes the PEE technique with ring prediction and incorporates a sorting strategy based on discrete mean curvature for smoothness evaluation, respectively.

**Fig. 6:**
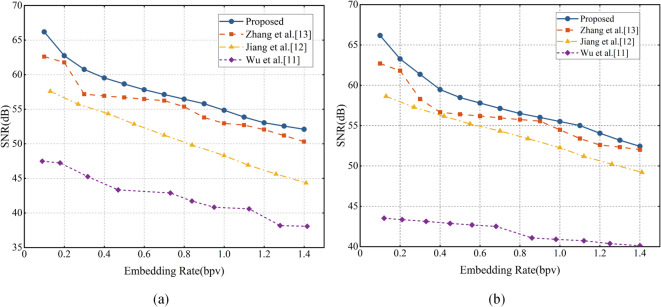
Performance of the proposed method compared with methods^11–13^ on (**a**) Bunny; (**b**) Horse.

Firstly, the experiment results obtained from the Bunny and Horse models are shown in Fig. 6. We can see that the proposed method achieves a higher SNR at given embedding rates and consistently outperforms other three methods. This demonstrates that the proposed method introduces a lower geometric distortion after data embedding. Compared with the conventional PEE method^11^, the proposed method performs better by a large margin, with a maximum SNR gain of 22.65 dB. The conventional PEE method neglect the embedding sequence. As an early PEE method in RDH for 3D mesh models,^11^ has a great potential for improvement. Compared with Jiang et al.’s PEH modification method^12^, the proposed spatial polygon prediction scheme increases the number of reference vertices for prediction over the odd–even prediction scheme, thus the proposed method obtains a better prediction accuracy and achieves higher SNR. Compared with Zhang et al.’s PEE with sorting method, which has been verified as an efficient RDH method, our method can outperform it across different embedding rates. Compared with the methods in^11–13^, the proposed method yields a significant improvement in average SNR by 15.20 dB, 5.19 dB, and 1.69 dB at the same embedding rate, respectively.

**Fig. 7:**
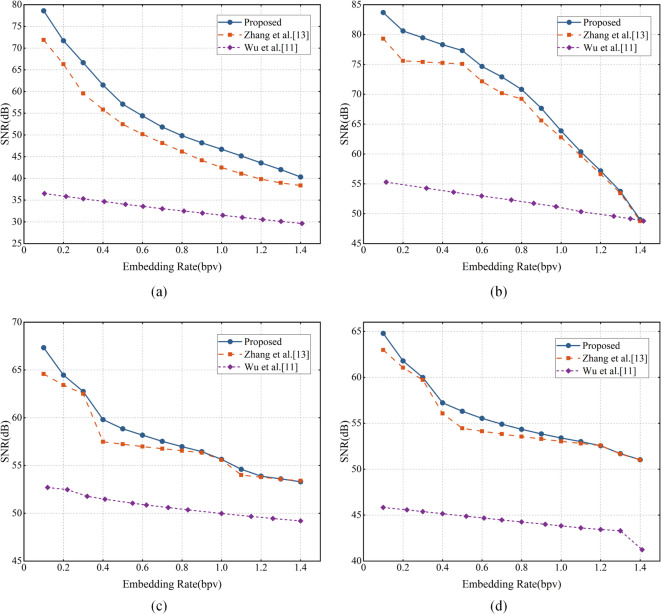
Performance of the proposed method compared with method^11,13^ on (**a**) Casting; (**b**) Crank; (**c**) Dragon; (**d**) Venus.

In addition, the proposed method is also compared with Wu et al.’s conventional PEE method^11^ and Zhang et al.’s PEE with sorting method^13^ on four additional models: Casting, Crank, Dragon, and Venus. As shown in Fig. 7, our proposed method consistently achieves superior performance in geometric fidelity. Taking the Casting model as an example, at an embedding rate of 0.6 bpv, the proposed method achieves an SNR of 54.39 dB. This compares favorably to the 33.56 dB achieved by^11^ and the 50.18 dB by^13^. These results demonstrate that the performance enhancement is not model-specific but a reliable characteristic of our method.

To quantitatively evaluate the performance gains, we conducted experiments on six models (Bunny, Horse, Casting, Crank, Dragon, and Venus) to summarize the average SNR improvements achieved by method^11^ and method^13^. As shown in Table 3, the proposed method consistently outperforms the comparison methods across all six test models in terms of average SNR gain under the same embedding rate. Compared with method^11^, the proposed method achieves an average SNR improvement of 18.46 dB, with individual gains ranging from 9.71 dB (Dragon) to 28.40 dB (Casting). This substantial improvement can be attributed to the fact that method^11^ lacks sorting strategy, which are essential for reducing prediction errors and embedding distortion. In contrast, when compared with method^13^, which incorporates two-layer division and ring prediction, the proposed method still achieves a notable average SNR gain of 2.62 dB, with gains ranging from 1.19 dB (Venus) to 5.34 dB (Casting). This improvement reflects the advantages of the proposed three-layer vertex division, spatial polygon prediction, and dual sorting strategy, which enable large embedding capacity and finer smoothness evaluation, thereby reducing geometric distortion more effectively. The consistent gains further validate the generalizability of the proposed method.

**Table 3:** Average SNR gains (dB) compared with methods^11^ and^13^.

Comparison method	Bunny	Horse	Casting	Crank	Dragon	Venus
^11^	+ 16.30	+ 17.79	+ 28.40	+ 25.08	+ 9.71	+ 13.50
^13^	+ 2.13	+ 2.23	+ 5.34	+ 3.42	+ 1.42	+ 1.19

To evaluate the computational performance of the proposed method, we compared it with methods^11^ and^13^ under identical hardware and software configurations. All eight standard models were tested at a fixed embedding rate of 0.7 bpv. As shown in Table 4, owing to its dual sorting strategy, the proposed method incurs a slightly higher runtime than^13^ (which adopts a single sorting strategy), but remains significantly faster than^11^. Specifically, for the Bunny model (with 61, 154 vertices), the runtime of the proposed method is approximately 1.87 s, compared to 1.56 s for^13^ and 4.25 s for^11^. The average runtimes across all eight models are 13.89 s for^11^, 4.53 s for^13^, and 5.46 s for the proposed method. For large-scale meshes such as the Dragon model (with 437, 645 vertices), the runtime is 24.01 s, indicating that the proposed method can complete the process within several tens of seconds even for million-vertex meshes. These results demonstrate that the additional computational overhead introduced by dual sorting is acceptable, and the overall efficiency of the proposed method remains competitive.

**Table 4:** Runtime comparison of different methods (in seconds).

Method	Bunny	Horse	Casting	Crank	Dragon	Venus	Dinosaur	Hand
^11^	4.25	4.54	9.49	13.70	44.97	9.36	3.79	20.97
^13^	1.56	2.12	0.29	2.21	19.91	6.00	2.48	1.70
proposed	1.87	2.64	0.32	2.65	24.01	7.17	2.93	2.11

### Performance evaluation of sorting strategies

To further validate the superiority of the dual sorting strategy, we compare it with Zhang et al.’s PEE with sorting method^13^ on four mesh models: Bunny, Horse, Casting, and Crank. The proposed dual-sorting strategy jointly considers angular smoothness and edge-length regularity, while^13^ relies solely on discrete mean curvature as its smoothness criterion. As shown in Table 5, the average SNR gains for the Bunny, Horse, Casting, and Crank models are 2.67 dB, 2.48 dB, 5.37 dB, and 3.54 dB, respectively. The proposed method achieves an average SNR gain of 3.51 dB, surpassing Zhang et al.’s PEE with sorting method^13^ in all test cases, with a peak gain of 7.27 dB on the Casting model at 0.28 bpv. The dual sorting strategy plays a key role in boosting performance, as it selects smooth embedding units where vertex prediction is accurate and PEs are small. Experimental results prove that the dual sorting strategy effectively enhances embedding performance.

**Table 5:** SNR(dB) performance of sorting strategies across different ER (bpv).

Model	Method	0.06	0.13	0.21	0.28	0.34	0.41	0.48	0.56	0.63	0.70
Bunny	^13^	62.99	62.35	58.84	57.23	57.09	56.91	56.73	56.54	56.40	56.24
	Proposed	68.24	64.96	62.45	60.98	60.25	59.43	58.83	58.12	57.59	57.14
Horse	^13^	63.13	62.41	61.72	58.39	56.81	56.64	56.47	56.28	56.12	55.96
	Proposed	68.04	64.96	63.03	61.69	60.32	59.37	58.62	58.04	57.57	57.13
Casting	^13^	74.15	70.24	65.32	60.57	57.92	55.42	52.93	50.94	49.51	48.15
	Proposed	78.09	76.91	71.35	67.84	64.99	61.00	57.90	55.40	53.53	51.84
Crank	^13^	79.48	79.17	75.58	75.45	75.35	75.23	75.10	73.32	71.04	70.17
	Proposed	85.07	82.47	80.48	79.76	79.06	78.15	77.52	75.98	73.86	72.90

To verify the individual contributions and synergistic effects of the two subcriteria in the dual sorting strategy, we conducted an ablation study comparing three configurations: sorting based solely on angular smoothness (*AS*), sorting based solely on edge-length regularity (*ELR*), and the combined dual sorting parameter (*DSP*) strategy. Experiments were performed on the Bunny model across various embedding rates, with the corresponding SNR results summarized in Table 6. The experimental results demonstrate that each individual criterion achieves satisfactory embedding performance, while their combination in the dual sorting strategy further improves the SNR, with gains of up to 1.3 dB at low embedding rates and approximately 0.5 dB at high embedding rates. This confirms that *AS* and *ELR* in the dual sorting strategy complement each other, enabling a more comprehensive characterization of the embedding unit smoothness and thus more effectively reducing geometric distortion.

**Table 6:** Internal ablation study on dual sorting strategy.

Method	0.06	0.13	0.21	0.28	0.34	0.41	0.48	0.56	0.63	0.70
AS	66.91	63.83	61.97	60.49	59.55	58.74	58.12	57.53	57.09	56.68
ELR	67.09	64.48	62.30	60.73	59.83	59.12	58.49	57.88	57.39	56.95
DSP	68.24	64.96	62.45	60.98	60.25	59.43	58.83	58.12	57.59	57.14

## Discussion and potential extensions

### Robustness analysis

It is worth noting that the RDH method proposed in this paper falls into the category of fragile watermarking, with its primary application scenarios being copyright marking, integrity authentication, and content annotation in lossless transmission environments. Unlike robust watermarking, fragile watermarking is highly sensitive to any form of carrier modification, which enables effective detection of malicious tampering but also implies that it cannot resist common mesh processing operations such as simplification, filtering, or geometric attacks. Therefore, when applying this method to practical systems, it is essential to ensure the losslessness of the transmission and storage channels or to integrate it with other robustness-enhancing techniques.

### Applicability to different 3D data formats

The proposed method is originally designed for manifold triangular meshes, yet its applicability can be extended to polygonal meshes. The adopted spatial polygon prediction scheme fundamentally relies on the adjacency relationships among vertices rather than the specific shape of triangles. For polygonal meshes (e.g., quadrilateral meshes), although each face contains multiple vertices, the definition of the 1-ring neighborhood—i.e., all vertices sharing an edge with the central vertex—remains valid. Therefore, the proposed method can be directly applied to arbitrary polygonal manifold meshes without any modification, simply by constructing polygons based on the neighboring vertices.

For more complex 3D data formats, such as non-manifold meshes (containing hanging edges or non-manifold vertices), point clouds (lacking topological connectivity information), and irregular topologies (e.g., T-junctions), the proposed method cannot be directly applied due to the absence of a stable 1-ring neighborhood structure. Nevertheless, in practical engineering applications, these data types can be converted into a compatible format through the following preprocessing strategies:

1. Non-manifold meshes: Mesh cleaning operations, including the removal of non-manifold edges, separation of non-manifold vertices, or local remeshing, can be employed to convert them into manifold meshes. Although such operations may slightly alter the original geometry, they are generally acceptable as preprocessing steps in most application scenarios.
2. Point clouds: Surface reconstruction algorithms, such as Poisson reconstruction or Delaunay triangulation, can be used to generate triangular mesh models. This approach is a conventional technique in the fields of computer graphics and geometric processing, and the reconstructed meshes can serve as valid inputs to the proposed method.
3. Irregular topologies: For structures such as quadrilateral meshes with T-junctions, topological regularization can be achieved through subdivision or conversion to triangular meshes.

### Potential extensions to other geometric processing tasks

This section discusses the potential applicability of the proposed spatial polygon prediction and dual sorting mechanisms to a broader range of 3D geometric processing tasks. Although the current method is specifically designed for reversible data hiding, its core ideas—leveraging local geometric structures for accurate prediction and prioritizing processing units based on geometric smoothness—exhibit a degree of generality that may be transferable to other tasks.

#### 3D mesh restoration and enhancement

Inspired by recent advances in deep learning-based image and video restoration, such as ESTINet^29^, which achieves video deraining through spatio-temporal interaction learning, and MC-Blur^30^, which provides a large-scale benchmark dataset for image deblurring, we observe that the proposed spatial polygon prediction mechanism has the potential to serve as a geometric prior in 3D mesh restoration and enhancement networks. For instance, in mesh denoising^31^ tasks, local polygon prediction can be leveraged to generate clean vertex positions as reference signals. In mesh completion tasks, the polygon prediction scheme can provide geometric constraints for missing regions. The proposed prediction method essentially estimates the central vertex by exploiting the geometric correlations among neighboring vertices, which aligns conceptually with the local feature extraction principles commonly employed in many learning-based approaches.

#### Transferability of the dual sorting strategy

The dual sorting strategy proposed in this work prioritizes embedding units based on angular smoothness and edge-length regularity, giving precedence to geometrically regular regions. This concept can be extended to tasks such as progressive mesh transmission and adaptive mesh simplification, where geometrically regular regions can be transmitted or preserved first, thereby improving efficiency while maintaining visual quality. Furthermore, in learning-based mesh processing frameworks, the proposed sorting strategy could be utilized as a sample weighting mechanism or a training sampling strategy, guiding models to focus more on geometrically complex regions.

## Conclusions

RDH for 3D models serves critical roles in various applications, such as copyright protection, integrity authentication and content annotation. In this paper, a RDH method for 3D mesh models based on spatial polygon prediction and dual sorting is proposed. A three-layer vertex division mechanism is employed to support a two-round embedding process, which increases the number of embedded vertices and achieves higher embedding capacity. The spatial polygon prediction scheme achieves a balance between prediction accuracy and embedding capacity. By prioritizing the smoother embedding units for data embedding, the dual sorting strategy significantly reduces the geometric distortion. Experimental results demonstrate that the proposed method achieves a more efficient capacity-distortion trade-off compared with the state-of-the-art methods.

Notwithstanding its competitive performance, the dual sorting strategy introduces a certain degree of computational cost, which represents a primary limitation and a clear direction for future work. To improve practical applicability, we will explore the design of more efficient approximate sorting algorithms (e.g., partitioned parallel sorting) in subsequent work. The goal is to reduce time complexity while maintaining or even improving overall performance. Successfully addressing this challenge would not only advance the technical framework presented here but also facilitate the adoption of RDH in real-time or large-scale 3D model applications.

Furthermore, the current method focuses on fragile reversible data hiding and does not consider robustness against mesh processing operations. In the future, it is worth exploring the integration of this method with robust watermarking techniques, such as introducing redundancy coding or spread spectrum techniques during the embedding stage. This would enable partial data extraction after mild processing operations (e.g., quantization, compression) or facilitate tamper localization when alterations are detected. This represents an important direction for enhancing the practical applicability of the method.

## Data Availability

Data sets generated during the current study are available from the corresponding author on reasonable request. The 3D models data that support the findings of this study are available from a Benchmark for 3D Mesh Watermarking. Data are available at https://projet.liris.cnrs.fr/meshben/ with the permission of the M2DisCo team of the LIRIS laboratory.
